# Androgenetic alopecia: new insights into the pathogenesis and mechanism of hair loss

**DOI:** 10.12688/f1000research.6401.1

**Published:** 2015-08-19

**Authors:** Rodney Sinclair, Niloufar Torkamani, Leslie Jones

**Affiliations:** 1 Department of Medicine, University of Melbourne, Victoria, Australia; 2 Epworth Dermatology, Victoria, Australia; 3 Sinclair Dermatology, Victoria, Australia

**Keywords:** Androgenetic, alopecia, follicle

## Abstract

The hair follicle is a complete mini-organ that lends itself as a model for investigation of a variety of complex biological phenomena, including stem cell biology, organ regeneration and cloning.

The arrector pili muscle inserts into the hair follicle at the level of the bulge- the epithelial stem cell niche.  The arrector pili muscle has been previously thought to be merely a bystander and not to have an active role in hair disease.

Computer generated 3D reconstructions of the arrector pili muscle have helped explain why women with androgenetic alopecia (AGA) experience diffuse hair loss rather than the patterned baldness seen in men.  Loss of attachment between the bulge stem cell population and the arrector pili muscle also explains why miniaturization is irreversible in AGA but not alopecia areata.

A new model for the progression of AGA is presented.

## Introduction

**Figure 1.**
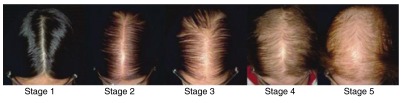
Sinclair scale for female pattern hair loss. Stage 1 is normal. Stage 2 shows widening of the central part. Stage 3 shows widening of the central part and loss of volume lateral to the part line. Stage 4 shows the development of a bald spot anteriorly. Stage 5 shows advanced hair loss.

Androgenetic alopecia (AGA) affects both genders and is characterised by hair loss in a distinctive and reproducible pattern from the scalp ^1^. Bitemporal recession affects 98.6% of men and 64.4% of women, whereas mid-frontal hair loss ( Figure 1) affects nearly two thirds of women over the age of 80 years, and three quarters of men over 80 years have mid-frontal and vertex hair loss ^2^. Local and systemic androgens transform large terminal follicles into smaller vellus-like ones ^3^. Follicular miniaturization is the histological hallmark of AGA ^4, 5^.

**Figure 2.**
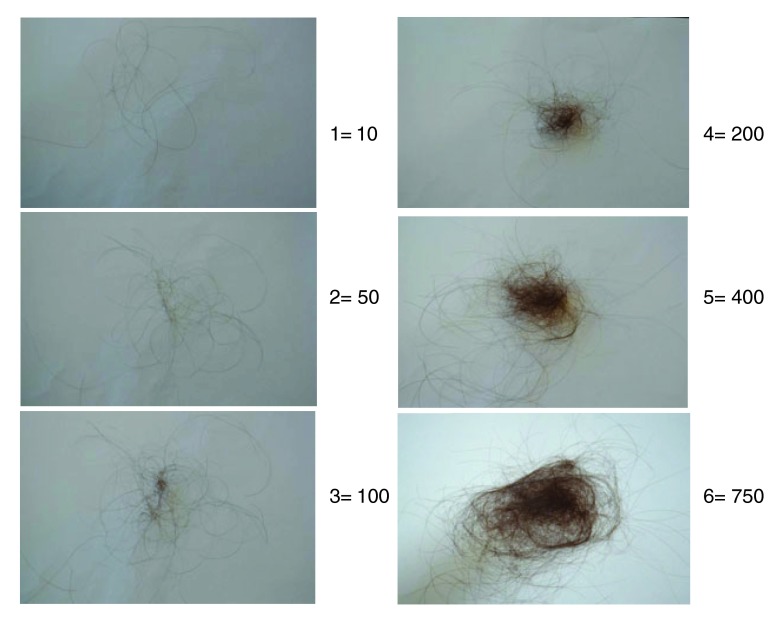
Hair shedding scale. Women are asked which image best corresponds to the amount of hair shed on an average day. Grades 1 to 4 are considered normal for women with long hair. Grades 5 and 6 indicate excessive shedding. Seventy percent of women with female pattern hair loss have excessive shedding.

Diffuse hair thinning and sometimes increased hair shedding ( Figure 2) precede the clinical appearance of baldness by a number of years ^6^. This is because the process of follicular miniaturization which occurs in AGA does not simultaneously affect all follicles within a follicular unit (FU). Instead, there is a hierarchy of follicular miniaturization within FUs, and secondary follicles are affected initially and primary follicles are miniaturized last ^7^.

## Histology of follicles in androgenetic alopecia

**Figure 3.**
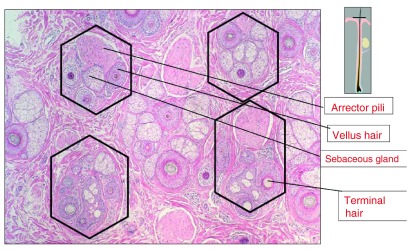
Horizontal section of skin biopsy from a hairy scalp showing features of early androgenetic alopecia. Follicles exist within follicular units comprising arrector pili muscle, sebaceous gland, and derived secondary hairs, some of which have miniaturized to become secondary vellus hairs. The image in the upper right depicts the level of the follicle where the horizontal sections have been cut.

**Figure 4.**
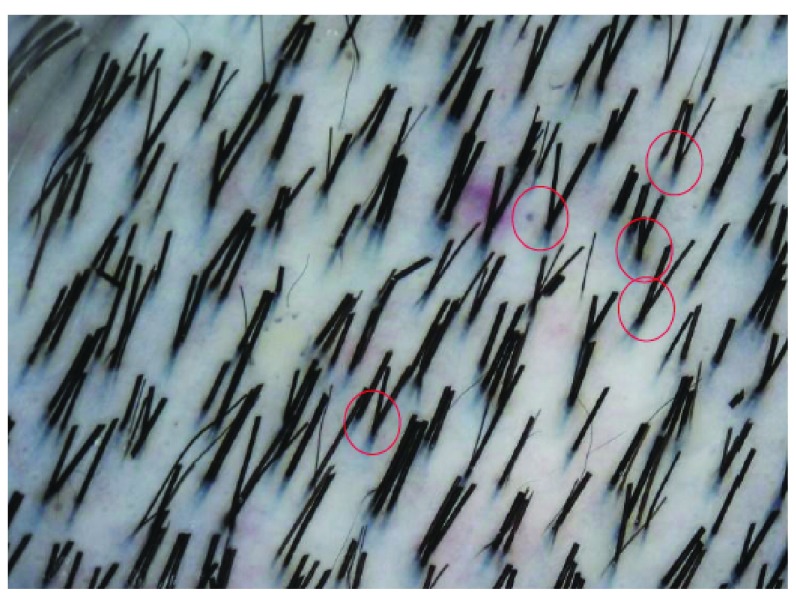
Normal scalp. Multiple hair fibres can be seen to emerge from a single infundibulum.

**Figure 5.**
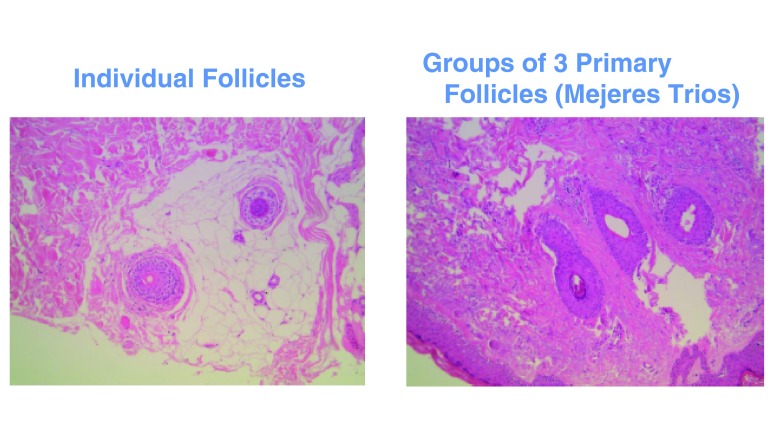
Horizontal section of skin biopsy from a hairy forearm showing follicles to exist singly or in groups of three, known as Mejeres trios.

**Figure 6.**
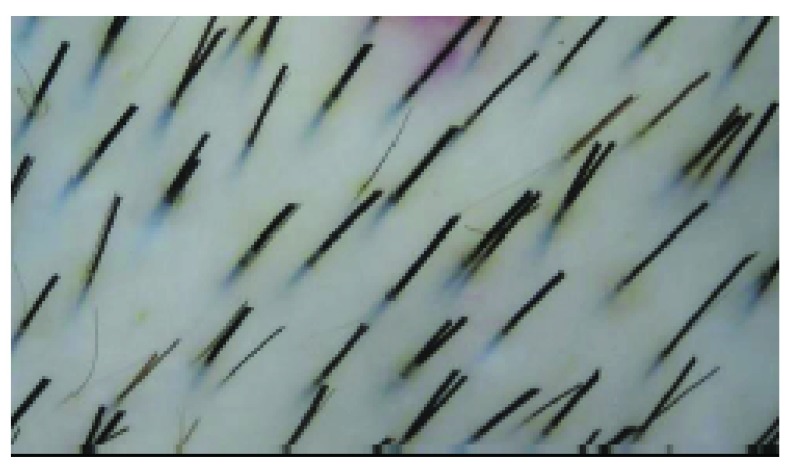
In androgenetic alopecia, a reduction in the number of hairs per follicular unit precedes the development of baldness.

Scalp hairs arise from FUs that are best seen on horizontal scalp biopsy. FUs comprise a primary follicle that gives rise to an arrector pili muscle (APM), a sebaceous gland, and multiple secondary follicles that arise distal to the APM ( Figure 3). Hairs from secondary follicles commonly emerge from a single infundibulum ( Figure 4). In contrast, hairs over the beard, trunk, and limbs do not give rise to secondary hairs and exist singly or in groups of three, known as Mejeres trios ( Figure 5). Miniaturization occurs initially in the secondary follicles, leading to the reduction in hair density that precedes visible baldness ( Figure 6). Baldness ensues when all of the hairs within an FU are miniaturized.

## Role of the arrector pili muscle: New findings and implications for androgenetic alopecia

One intriguing question is that identical hair follicle miniaturization is seen histologically in lesions of alopecia areata. In this condition, miniaturization of all follicles occurs simultaneously, and unlike AGA, miniaturization occurring in alopecia areata is potentially fully reversible.

**Figure 7.**
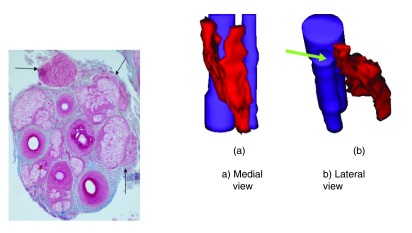
In telogen effluvium and also alopecia areata, the arrector pili muscle (red) can be shown to be attached to the hair follicle (purple). ( **a**) 3-dimensional reconstruction of the follicular unit with the muscles coloured red and follicles blue rotated to the left and ( **b**) to the right.

**Figure 8.**
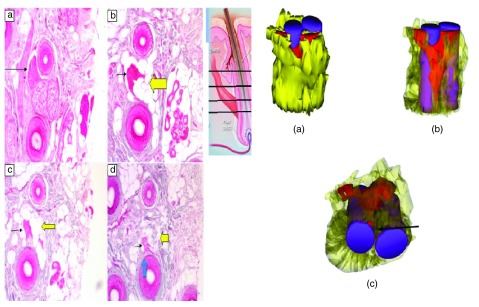
In androgenetic alopecia, the proximal arrector pili muscle (red) is progressively replaced by adipose tissue (yellow) and loses its attachment to the hair follicle bulge (purple).

This apparent paradox may be explained by examination of the APM and in particular its proximal attachment to the hair follicle bulge ^8^. The APM is a small band of smooth muscle that runs from the hair follicle to the adjacent upper dermis and epidermis. This muscle contributes to thermoregulation and sebum secretion. The APM arises proximally at the hair follicle at the bulge, which is an epithelial stem cell niche. Three-dimensional reconstructions of scalp biopsy specimens demonstrate that preservation of the APM predicts reversible hair loss ( Figure 7) and that, conversely, loss of attachment between the APM and hair follicle bulge is associated with irreversible or partially reversible hair loss ( Figure 8).

The APM plays a significant role in maintaining hair follicle integrity. Restoration of the APM in transplanted hair follicle units has been shown to induce the regeneration of the neurofollicular and neuromuscular junction in the follicle bulge in single FU transplants in patients with AGA ^9^.

The discovery that progressive muscle volume loss and fat infiltration of the APM leading to total or near total loss of the muscle attachment to the primary follicle bulge in AGA samples ^10^ led to the hypothesis that maintenance of the attachment between the APM and the bulge might differentiate between reversible and irreversible hair follicle miniaturization. These features were exclusive to AGA and not seen in alopecia areata, a disorder associated with reversible hair follicle miniaturization ^11^. The finding that the APM is preserved in telogen effluvium and alopecia areata supports this view.

It appears likely that the interaction between the mesenchyme-derived APM and the follicle bulge epithelium is essential for the integrity of the pilosebaceous unit, much in the same way as the interaction between the mesenchymal-derived dermal papilla and the epithelial hair follicle matrix.

**Figure 9.**
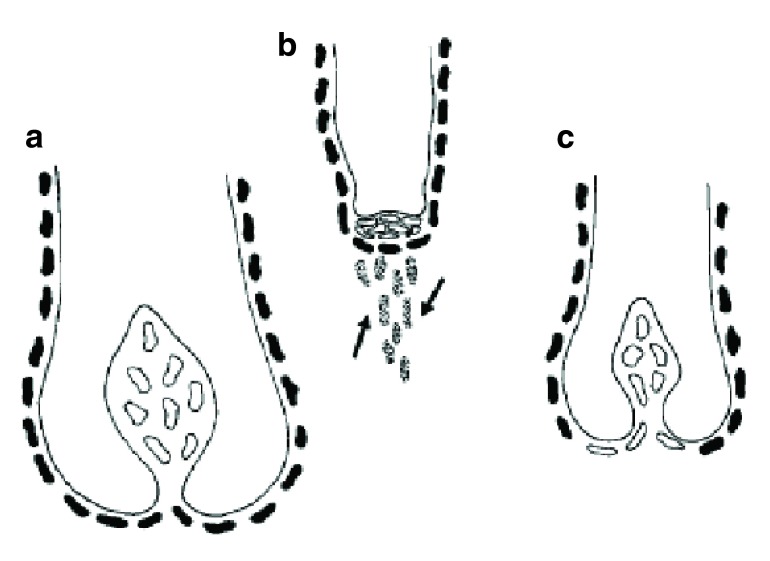
Reduction in dermal papilla cell numbers as an indirect result of changes to the dermal sheath. The sheath cells (solid cells) that surround the follicle are an integral part of the follicle dermis ( **a**). If they are functionally lost (dotted cells indicated by arrows) from the follicle ( **b**), then dermal papilla cells (outline only) move from the papilla to replace them ( **c**). As a result, the papilla and the follicle become smaller. Reproduced with permission from John Wiley & Sons, Inc. ^12^.

Follicle cycling is associated with the movement of cells between the dermal papilla and dermal sheath ^12^. It is thought that disruption of this process in AGA causes a loss of cells from the dermal sheath and then the dermal papilla that leads to hair follicle miniaturization ( Figure 9). Cells from the dermal papilla and dermal sheath are capable of undergoing both smooth muscle and adipose differentiation *in vitro*. Cells from the follicle mesenchyme might also contribute to maintenance of the APM, and the muscle degeneration seen in AGA could be caused by the loss of a progenitor cell population that maintains both the APM and the dermal papilla.

## Research summary

**Figure 10.**
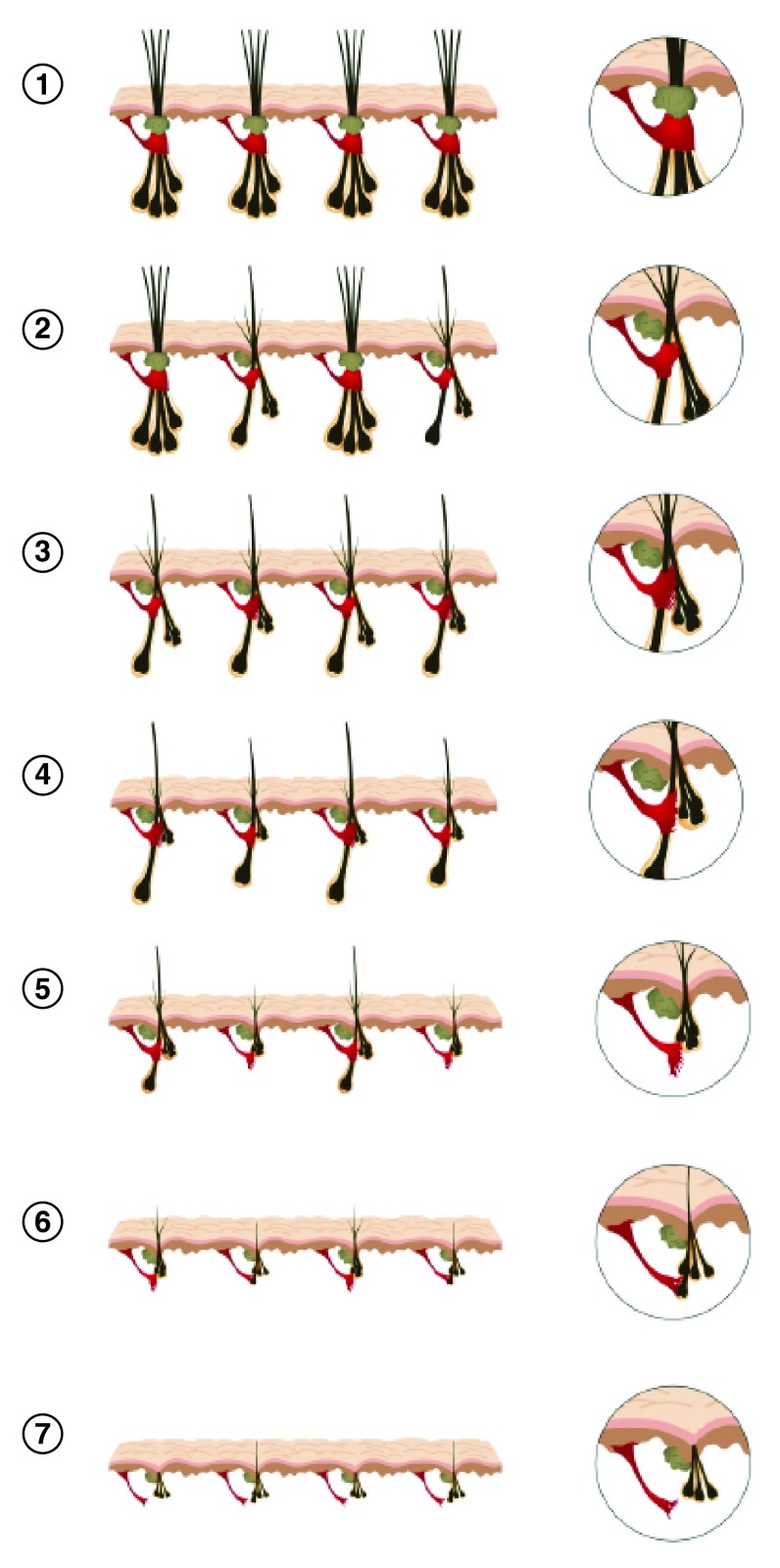
Scalp follicles exist as compound follicular units. In androgenetic alopecia, miniaturization occurs initially in the secondary follicles. This leads to a reduction in hair density that precedes visible baldness. Bald scalp becomes visible only when all of the hairs within a follicular unit are miniaturized. With miniaturization, the muscle initially loses attachment to the secondary follicles. When primary follicles eventually miniaturize and lose muscle attachment, the hair loss becomes irreversible.

In conclusion, we propose a new model for AGA ( Figure 10). In early stages of hair loss, the APM remains attached to the primary follicle but loses its attachment to some of the regressing secondary follicles in some FUs. Miniaturization of secondary follicles and detachment of the APM from these follicles extend to the rest of the FUs. At this stage, patients may complain of hair thinning and loss of volume in their pony tail without visible baldness.

With further progression, miniaturization continues and the muscle loses attachment to the secondary follicles in affected FUs completely. Primary follicles eventually miniaturize and this leads to visible baldness. When primary follicles lose muscle attachment, the hair loss becomes irreversible. Hopefully, this model facilitates a clearer understanding of normal physiological hair growth and also alterations to hair growth in hair loss conditions.
