# Supplementary material for: Androgenetic alopecia: new insights into the pathogenesis and mechanism of hair loss
Source: F1000Res. 2015 Aug 19;4(F1000 Faculty Rev):585. [Version 1] doi: 10.12688/f1000research.6401.1 (PMC4544386; doi:10.12688/f1000research.6401.1)
Supplement: Peer review history

# Peer review: Referee response for version 1

**DOI:** 10.5256/f1000research.6867.r10015

**Published:** 2015-08-19

**Authors:** Victoria Jolliffe

**Affiliations:** 1 Department of Dermatology, Royal London Hospital, London, UK

I have read this submission. I believe that I have an appropriate level of expertise to confirm that it is of an acceptable scientific standard.

# Peer review: Referee response for version 1

**DOI:** 10.5256/f1000research.6867.r10014

**Published:** 2015-08-19

**Authors:** Lynne Goldberg

**Affiliations:** 1 Department of Dermatology, Boston University School of Medicine, Boston, MA, USA

This review presents a novel concept that incorporates a three dimensional view into the understanding of androgenetic alopecia, and introduces the arrector pili muscle as a new player in the game.

I have read this submission. I believe that I have an appropriate level of expertise to confirm that it is of an acceptable scientific standard.

# Peer review: Referee response for version 1

**DOI:** 10.5256/f1000research.6867.r10013

**Published:** 2015-08-19

**Authors:** Matthew Harries

**Affiliations:** 1 Salford Royal NHS Foundation Trust, Salford, UK

I have read this submission. I believe that I have an appropriate level of expertise to confirm that it is of an acceptable scientific standard.

# Peer review: Referee response for version 1

**DOI:** 10.5256/f1000research.6867.r10008

**Published:** 2015-08-19

**Authors:** Satoshi Itami

**Affiliations:** 1 Department of Regenerative Dermatology, Osaka University, Suita, Japan

I have read this submission. I believe that I have an appropriate level of expertise to confirm that it is of an acceptable scientific standard.
